# Foxn1[Cre] Expression in the Male Germline

**DOI:** 10.1371/journal.pone.0166967

**Published:** 2016-11-23

**Authors:** Jianjun Shi, Irina Getun, Bivian Torres, Howard T. Petrie

**Affiliations:** Department of Immunology and Microbial Sciences, The Scripps Research Institute, Jupiter, FL, United States of America; National Eye Centre, UNITED STATES

## Abstract

Foxn1 (forkhead box N1), also known as the nude gene or winged-helix nude (Whn), is a forkhead transcription factor thought to be restricted to keratinocytes in the skin and thymus. Consistent with this tissue distribution, spontaneous or targeted mutation of Foxn1 results in the absence of both hair and a thymus. Genetic manipulation of the Foxn1 locus thus represents a powerful tool for tissue specific gene control in the skin and thymus, and tools such as Cre recombinase under control of the Foxn1 locus are widely used for this purpose. Unexpectedly, we show that Foxn1[Cre] exhibits unexpected activity in male germ cells, resulting in ubiquitous targeting of loxP-flanked alleles in all tissues in offspring from Foxn1[Cre] expressing male mice. Inheritance of recombined loxP alleles occurs independently of Cre inheritance (i.e., offspring lacking Cre nonetheless exhibit recombined alleles), suggesting that Foxn1[Cre] induced recombination in male germ cells must occur prior to meiosis in diploid germ cells. Together with previously published data, our results show that Foxn1, and alleles under its control, are expressed in the pre-meiotic male germline, revealing a new tool for germline targeting of genes, and raising important concerns for gender selection when using Foxn1 regulatory elements.

## Introduction

Conditional gene activation or gene deletion is a powerful contemporary tool for probing the function of encoded gene products. Most often, this approach involves homologous recombination of gene segments or regulatory elements in a tissue specific manner, using bacterial endonucleases such as Cre. Tissue specificity is induced by placing Cre under the control of a carefully chosen promoter that is specific for the tissue of interest. However, since most genes are expressed in multiple tissues, collateral targeting of conditional alleles in unintended tissues is a frequent concern.

Foxn1 is a forkhead transcription factor that has been shown to be responsible for the nude phenotype [1], a spontaneously derived mutation characterized by the absence of hair and thymic tissue [2]. Alleles of Foxn1 have therefore been extensively used to study the biology of hair, skin, and thymus. Activity of Foxn1[Cre] in other tissues has not been noted, although Foxn1 gene transcription has been reported to be more widespread than thymus and hair follicles/skin [3].

In this study, we show that Cre under the control of the Foxn1 locus results in conditional deletion of loxP-flanked alleles when carried in the male germline, but not the female. The offspring of Foxn1[Cre]-expressing males exhibit recombined loxP alleles, and activity of recombines alleles, in all tissues of the body. This occurs independently of whether Foxn1[Cre] was also inherited from the male parent, indicating that Cre-mediated recombination in the male germline must occur in pre-meiotic, diploid cells. Consequently, the effects of Foxn1[Cre]-mediated gene targeting are not tissue specific when this allele is carried by the male parent, but rather occur in all somatic tissues, independently of the inheritance of Cre.

## Materials and Methods

### Mice

Mice carrying a loxP-flanked Birc5 allele [4] were generously provided by Dr Pradip Roy-Burman (University of Southern California). Foxn1[Cre] knockin mice [5] were obtained from the Jackson Laboratories (strain 018448). Rosa[mTomato/mEgfp] mice [6] were obtained from the Jackson Laboratories (strain 007676). Mice were bred and maintained at Scripps after approval by The Scripps Institutional Animal Care and Use Committee (protocol 14–006). Euthanasia was conducted by CO_2_ inhalation, according to applicable NIH guidelines.

### Genotyping

Foxn1[Cre], Birc5^fl^, and Rosa[mTmG] were genotyped by PCR using tissue derived from toe clipping (for mouse identification). Note that in this tissue, consisting of both keratinocytes and cells of other lineages, both recombined and germline loxP-flanked alleles are expected when Foxn1[Cre] is present. Primer sequences were as follows:

Cre-forward, CGATGCAACGAGTGATGAGG
Cre-reverse, GCATTGCTGTCACTTGGTCG
Birc5(floxed)-forward, CTTGCCACGATGGTGATGAAACT
Birc5(floxed)-reverse, TCCTGTCAGAGAACACTGTCCCTT
Birc5(recombined)-forward, caggccgatggtctcagaaata
Birc5(recombined)-reverse, gcttaagtccacgtcacaatagagc
Rosa26-forward (reporter), CTCTGCTGCCTCCTGGCTTCT
Rosa26-reverse (reporter), TCAATGGGCGGGGGTCGTT.

Rosa[mTmG] PCR conditions were as follows. MgCl_2_ concentration was 2 mM. The first cycle was 94°C for 3’, followed by 35 cycles of (94°C for 30”, 61°C for 60”, and 72°C for 60”), and a final cycle of 72°C for 2’ prior to storage or agarose gel analysis.

Foxn1[Cre] PCR conditions were as follows. MgCl_2_ concentration was 2 mM. The first cycle was 94°C for 3’, followed by 35 cycles of (94°C for 30”, 60°C for 60”, and 72°C for 60”), and a final cycle of 72°C for 5’ prior to storage or agarose gel analysis.

Floxed/WT Birc5 PCR conditions were as follows. MgCl_2_ concentration was 1.5 mM. The first cycle was 94°C for 3’, followed by 35 cycles of (94°C for 30”, 68°C for 60”, and 72°C for 60”), and a final cycle of 72°C for 5’ prior to storage or agarose gel analysis.

Recombined Birc5 PCR conditions were as follows. MgCl_2_ concentration was 2 mM. The first cycle was 94°C for 3’, followed by 35 cycles of (94°C for 30”, 62°C for 30”, and 72°C for 60”), and a final cycle of 72°C for 5’ prior to storage or agarose gel analysis.

### Tissue sectioning and analysis

Tissues were harvested immediately after euthanasia and mounted in Tissue-Tek O.C.T. compound (VWR Scientific) for cryosectioning of 5 μm specimens onto SuperFrost Plus slides (ThermoFisher). Slides were stored desiccated at -20°C until used. Tissues for analysis were first fixed for 30 min in 2% EM-grade CH_2_O (Polysciences) in PBS on ice, followed by washing in ice-cold PBS and mounting using Prolong Gold with DAPI (Invitrogen). Image acquisition was performed by epifluorescence microscopy using a Lumen 200 metal halide light source.

### Flow cytometric analysis of white blood cells

30–50 μl of whole blood was collected from the retro-orbital plexus using heparin-coated capillary tubes, and diluted into Hanks’ balanced salt solution (HBSS) containing heparin (10 units/ml). After centrifugation, red blood cells were lysed by incubation in a solution consisting of 150 mM NH_4_Cl / 10 mM KHCO_3_ / 0.1 mM EDTA, followed by centrifugation through an underlay of 100% fetal bovine serum (FBS). The white blood cell pellet was resuspended in HBSS containing 5% FBS, 10 μg/ml of DNAse I, and DAPI at 50 ng/ml. Data was collected using an LSR2 cytometer (BD Biosciences) fitted with 405 nm, 488 nm, and 561 nm lasers (DAPI, GFP, or tdTomato, respectively). Dead (DAPI staining) cells were excluded from the analysis.

## Results

### Detection of recombined loxP alleles in Cre-negative offspring from Foxn1[Cre]-positive male, but not female, mice

**Fig 1:**
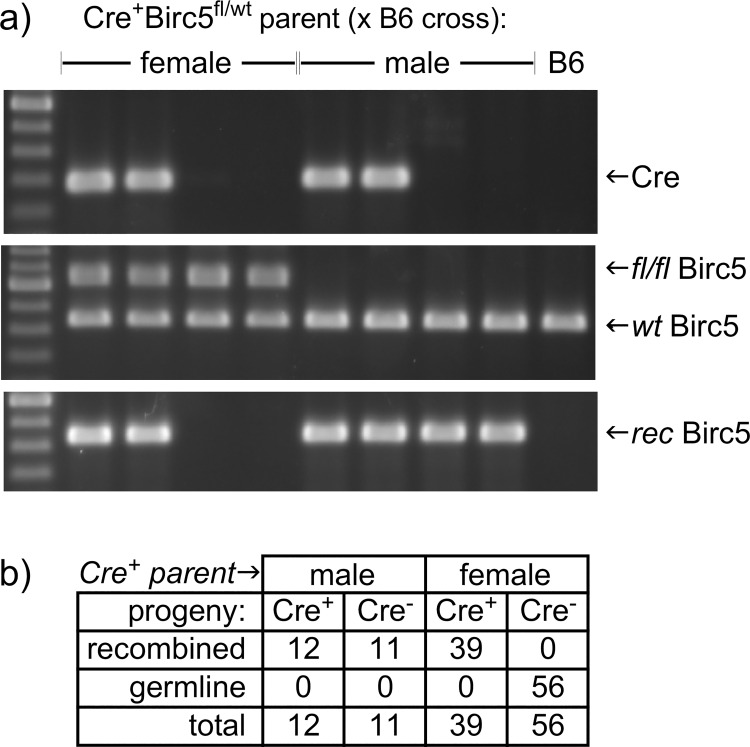
Recombined alleles appear independently of Cre expression in offspring from Foxn1[Cre]+ male, but not female, mice. a) PCR gel images using primers specific for Cre (top), primers that distinguish loxP-flanked (fl/fl) from wildtype (wt) alleles (middle), or primers that detect a recombined Birc5 allele (bottom), using DNA template from skin. When the female parent carried both Cre and the Birc5 conditional allele, only Cre+ offspring exhibited a recombined allele, as expected. However, offspring of the equivalent male parent showed recombination of Birc5 even in the absence of Cre. b) a summary of offspring from multiple independent litters.

During a study where Foxn1-driven Cre recombinase [5] was used to delete a loxP-flanked Birc5 gene [4] in thymic epithelial stromal cells, we made the observation that Cre-negative offspring unexpectedly exhibited the presence of rearranged Birc5^fl^ alleles. We established colony specifically to further test this observation (Fig 1). We found that offspring derived from female Foxn1[Cre] mice (crossed to C57BL/6 males) were genotyped in the expected ratios, exhibiting a recombined Birc5 allele only when Foxn1[Cre] has also been inherited (note: DNA from skin, which was the tissue used as a template for genotyping, includes both keratinocytes that express Foxn1 and other cell types that do not, and thus both a recombined allele and a floxed allele are detected when Cre is expressed). This was not the case, however, when male Foxn1[Cre] mice were crossed to C57BL/6 females. Instead, all offspring exhibited a recombined loxP allele, independent of whether they also inherited Cre. Importantly, even in Foxn1 non-expressing or mixed tissues (such as skin), a non-recombined allele was absent, suggesting that recombination of the floxed allele had occurred not only independent of transmission of Foxn1[Cre], but also prior to divergence of epithelial and mesenchymal (or other) tissue lineages.

### Inappropriate activation of a reporter allele, independent of Foxn1[Cre] inheritance, in the progeny of Foxn1[Cre] male, but not female, mice

**Fig 2:**
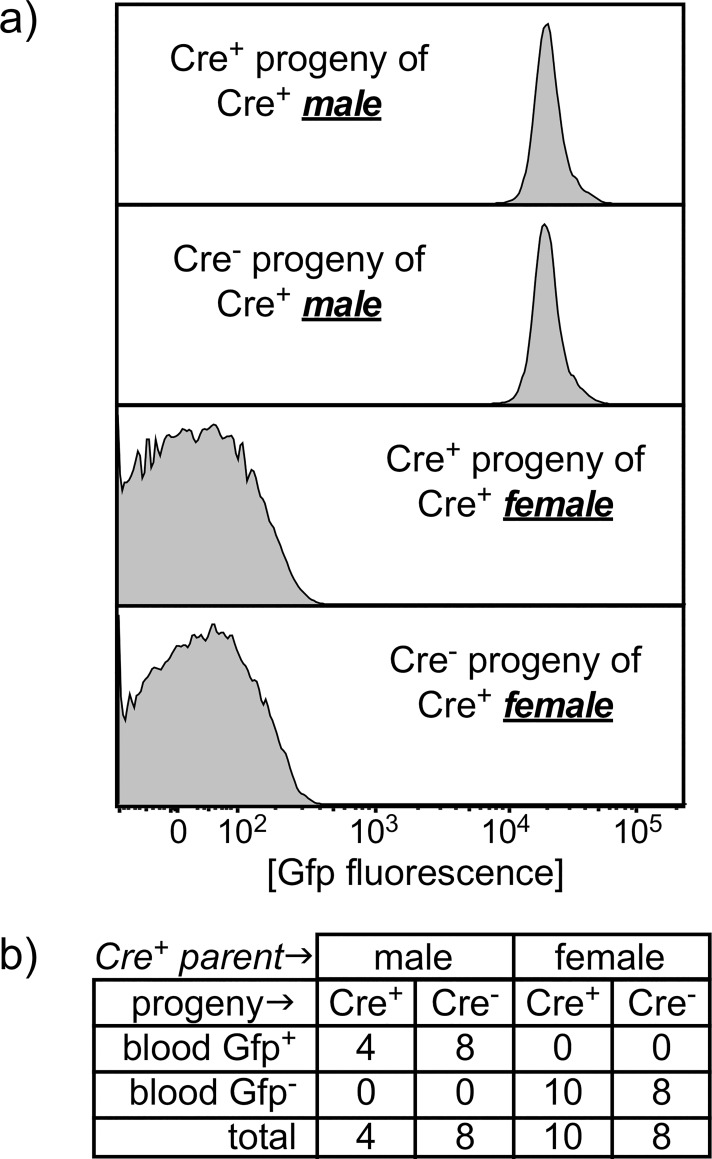
Inappropriate reporter activity, independent of Cre expression, in offspring from Cre+ male, but not female, mice. a) a conditional Gfp reporter expression in white blood cells (WBC) from Cre+ or Cre- offspring of Cre+ male or female parents (x B6 cross). WBC are uniformly Gfp+ when the male parent was Cre+, regardless of whether the offspring inherited Cre. Expression of a Foxn1[Cre]-activated conditional reporter is not expected in WBC from either Cre+ or Cre- offspring, since Foxn1 is not expressed in blood cells, as is the case when Foxn1[Cre] was carried by the mother. b) a summary of larger numbers of offspring from multiple independent litters.

To independently test this observation with a different conditional allele, and to examine functional activity rather than inheritance, we performed similar crosses of Foxn1[Cre] male or female mice to a Rosa26 double-fluorescent Cre reporter knockin mice [6] that express membrane-targeted Tomato Red fluorescent protein (mT) prior to Cre-mediated recombination, or membrane-targeted green fluorescent protein (mG) after recombination. As shown in Fig 2, the Cre-activated mG reporter was detected in white blood cells from the offspring of Foxn1[Cre] male X Rosa[mTmG] crosses regardless of whether Foxn1[Cre] was also inherited. Note that white blood cells do not normally express Foxn1 (or derive from Foxn1-expressing precursors), so Gfp expression is not expected in white blood cells whether Foxn1[Cre] is present or not; this is the case when Foxn1[Cre] females are crossed to Rosa[mTmG] males.

### Ubiquitous expression of a recombined reporter allele by the Cre-negative offspring of Foxn1[Cre]-expressing males

**Fig 3:**
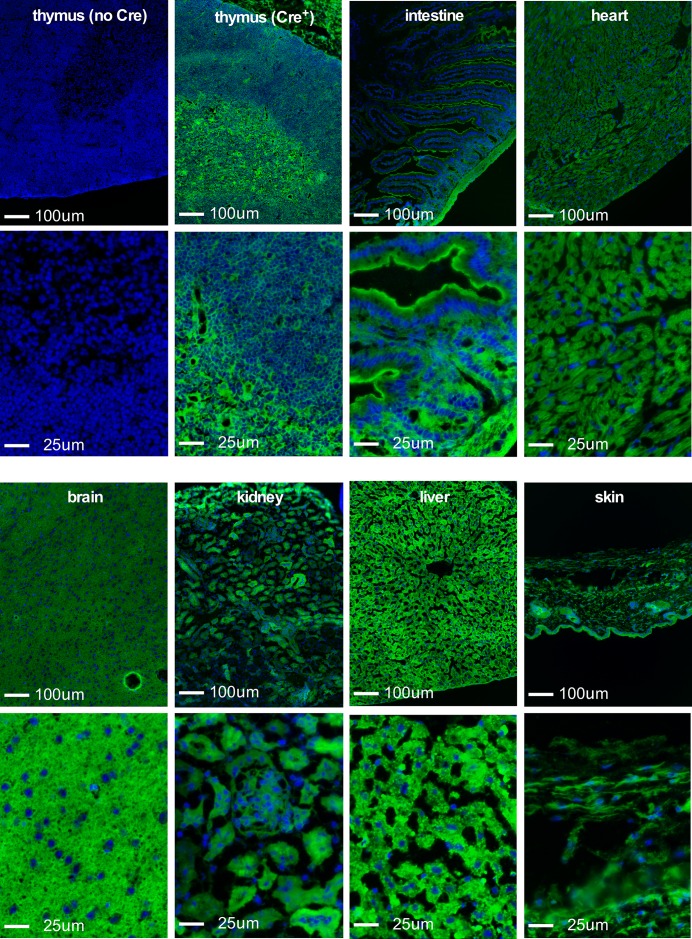
A conditional reporter is activated in all cells and tissues in Cre- offspring of Cre+reporter+ male x C57BL/6 female breeding. A conditional reporter is activated in all cells and tissues in Cre- offspring of Cre^+^reporter^+^ male x C57BL/6 female breeding. For each tissue, a 10x (top) or 40x (bottom) perspective is shown. Top left is thymic tissue from a conditional reporter mouse (Rosa26 knock-in stop/floxGfp) without Cre. DAPI (blue) indicates nuclei. All other panels are tissues from Cre- offspring of a male Foxn1[Cre]^+^Rosa[stop/floxGfp]^+^ x C57BL/6 female cross. All panels have the same exposure time for DAPI or for Gfp (at each magnification). All cells were Gfp^+^ in all tissues examined. Representative results are shown; the results were confirmed in tissues from 2–5 individual mice, depending on the tissue used.

The above findings suggest that Cre expression under the control of the Foxn1 locus results in promiscuous recombination of conditional loxP alleles when Cre is carried by the male parent, independent of Cre inheritance or Foxn1 expression in somatic tissue. To extend these findings, we examined a panel of tissues derived from Cre-negative offspring of Foxn1[Cre] male X Rosa[mTmG] female crosses. Fig 3 shows representative results from thymus, intestine, heart, brain, kidney, liver, or skin from a Foxn1[Cre]-negative Rosa[mTmG]-positive mouse; the activated Gfp reporter was seen in all cells in all tissues examined. This was not the case in Foxn1[Cre]-negative Rosa[mTmG]-positive offspring of female mice carrying the Foxn1[Cre] gene (not shown), as expected. Together, these findings substantiate our conclusion that Cre expression directed by Foxn1 regulatory sequences occurs in diploid male germ cells, but not in the female germline.

## Discussion

Foxn1 expression is believed to be restricted to keratinocytes in the skin and epithelial stromal cells in the thymus, consistent with the alopecia and athymia seen in naturally occurring or induced models of Foxn1 mutation [1]. Consequently, conditional expression using Foxn1 promoter-driven constructs is believed to target expression to skin and thymic tissues only. We unexpectedly found that when the Foxn1[Cre] allele is carried by the male parent, all offspring display recombined loxP alleles in all tissues, regardless of whether Foxn1 is normally expressed in that tissue and, importantly, regardless of whether Foxn1[Cre] was inherited by the same offspring. Since the Foxn1[Cre] strain that we used [5] is a bicistronic knockin of the Foxn1 locus that leaves endogenous Foxn1 otherwise intact, we expect that our results reflect true Foxn1 expression in male germ cells, rather than an artifact of the introduction of an exogenous gene (Cre). There are several other lines of evidence supporting the general nature of Foxn1 expression in testis. First, previously published studies have shown that Foxn1 mRNA is readily detected in mRNA template isolated from testis [3]. Second, testis from the naturally occurring Foxn1 mutant (and athymic) mouse strain designated nude [2] exhibit a variety of morphological abnormalities, including degeneration of both germ cells and epithelial cells [7]. Finally, an independent Foxn1-driven Cre transgene [8] also displays activity in the germline (Thomas Boehm, personal communication), albeit at lower penetrance and in both males and females, differences that are most likely due to epigenetic differences between the site of random transgene insertion vs. regulation of the endogenous Foxn1 locus.

At least two important considerations arise from our data. First is that Foxn1, and constructs regulated by this locus, joins an expanding list of genes expressed in tissues where they were not generally expected, often including the male germline, see [9,10–12] for examples. Expression of Foxn1[Cre] in the male germline can be a useful attribute, since it facilitates generation of knockout sperm in males heterozygous for genes that are lethal when homozygous. For instance, we used Foxn1[Cre]^+^ Birc5^fl/wt^ males crossed to a Birc5^fl/fl^ females to generate mice with one germline knockout allele of Birc5 and one allele that is conditionally targeted by Foxn1[Cre] in the skin and thymus only in Foxn1[Cre]^+^ offspring. However, the downside of unexpected Foxn1 activity in diploid male germ cells is that a targeted allele can be inherited even in the absence of Foxn1[Cre] expression (see Figs 1 and 3), an unpredicted outcome that may be easily overlooked in many cases. Further, germline activation by Foxn1[Cre] leads to expression in all tissues (Figs 2 and 3), not just those where Foxn1 is normally expressed (thymic epithelial cells, skin keratinocytes, and male germ cells). Consequently, mice derived from Foxn1[Cre]-positive male parents will display the conditional phenotype in all tissues, including Foxn1-negative tissues, even in the absence of Cre inheritance, thus confounding any interpretation where Cre-negative offspring of Foxn1[Cre]-positive males are used as controls. Although Cre expression in the female parent is frequently avoided because of occasional off-target effects, genetic models regulated by the Foxn1 locus are among those where expression in the female parent is the only option leading to tissue-specific results.
